# Inhibiting AGS Cancer Cell Proliferation through the Combined Application of Aucklandiae Radix and Hyperthermia: Investigating the Roles of Heat Shock Proteins and Reactive Oxygen Species

**DOI:** 10.3390/antiox13050564

**Published:** 2024-05-03

**Authors:** Chae Ryeong Ahn, In Jin Ha, Jai-Eun Kim, Kwang Seok Ahn, Jinbong Park, Seung Ho Baek

**Affiliations:** 1 College of Korean Medicine, Dongguk University, 32 Dongguk-ro, Ilsandong-gu, Goyang-si 10326, Republic of Korea; 2 Korean Medicine Clinical Trial Center (K-CTC), Korean Medicine Hospital, Kyung Hee University, Seoul 02447, Republic of Korea; 3 College of Korean Medicine, Kyung Hee University, 24 Kyungheedae-ro, Dongdaemun-gu, Soeul 02447, Republic of Korea

**Keywords:** Aucklandiae Radix, cancer, heat shock proteins, hyperthermia, reactive oxygen species

## Abstract

Cancer is a major global health concern. To address this, the combination of traditional medicine and newly appreciated therapeutic modalities has been gaining considerable attention. This study explores the combined effects of Aucklandiae Radix (AR) and 43 °C hyperthermia (HT) on human gastric adenocarcinoma (AGS) cell proliferation and apoptosis. We investigated the synergistic effects of AR and HT on cell viability, apoptosis, cell cycle progression, and reactive oxygen species (ROS)-dependent mechanisms. Our findings suggest that the combined treatment led to a notable decrease in AGS cell viability and increased apoptosis. Furthermore, cell cycle arrest at the G2/M phase contributed to the inhibition of cancer cell proliferation. Notably, the roles of heat shock proteins (HSPs) were highlighted, particularly in the context of ROS regulation and the induction of apoptosis. Overexpression of HSPs was observed in cells subjected to HT, whereas their levels were markedly reduced following AR treatment. The suppression of HSPs and the subsequent increase in ROS levels appeared to contribute to the activation of apoptosis, suggesting a potential role for HSPs in the combined therapy’s anti-cancer mechanisms. These findings provide valuable insights into the potential of integrating AR and HT in cancer and HSPs.

## 1. Introduction

Cancer remains a health challenge and is a leading cause of mortality worldwide, necessitating the development of effective and innovative treatment strategies [1,2,3,4]. Recently, the therapeutic paradigm has shifted towards a combination of different modalities to enhance treatment efficacy and potentially reduce associated side effects [5,6,7,8]. In particular, the integration of traditional herbal medicine with alternative therapeutic approaches such as hyperthermia (HT) has gained significant attention [9,10,11].

Aucklandiae Radix (AR), also known as Muxiang in traditional Chinese medicine, is a well-known medicinal herb with a rich history of therapeutic usage [12,13,14,15]. Extracted from the root of *Aucklandia lappa* DC., AR has been extensively used in various traditional medicinal systems for its broad-spectrum therapeutic properties [16,17,18]. Applications include the treatment of gastrointestinal disorders, alleviation of pain, and mitigation of inflammatory responses [19]. Among the diverse bioactive constituents of AR, costunolide and dehydrocostus lactone stand out for their potent anti-cancer activities [20,21,22]. Both compounds belong to the sesquiterpene lactone family; a class of compounds recognized for their cytotoxic effects against a variety of cancer cells [23,24]. Previous studies have suggested that the anti-cancer effects of these compounds are primarily mediated by the induction of reactive oxygen species (ROS).

ROS includes free radicals such as superoxide and hydroxyl radicals, and non-radicals such as hydrogen peroxide. ROS plays dual roles in cancer biology [25]. Under physiological conditions, ROS contribute to cellular homeostasis by modulating cellular signaling pathways [26]. However, when overproduced or insufficiently neutralized, ROS can induce oxidative stress, leading to cellular damage, apoptosis, and autophagy, making ROS generation a common target for anti-cancer strategies [27]. Costunolide and dehydrocostus lactone have been found to provoke a surge in ROS within cancer cells, thus tipping the balance towards apoptosis, the programmed cell death process that is often dysfunctional in cancer cells [28]. By triggering ROS-induced apoptosis, these compounds can effectively counteract the unchecked proliferation of cancer cells, which is a hallmark of cancer.

HT, the targeted application of heat to the body tissues, is emerging as an effective adjuvant therapy for cancer treatment [29,30,31]. Increasing the temperature of cancer cells increases their susceptibility to other anti-cancer therapies [32]. Mild HT, specifically around 43 °C, has shown promising results in combination with other treatments.

Heat shock proteins (HSPs) are intracellular proteins whose expression increases in response to stresses such as heat [33]. In the context of cancer, HSPs play critical roles in cell survival, proliferation, and therapeutic resistance [34,35]. They also help in maintaining intracellular homeostasis by preventing the accumulation of harmful ROS [36]. However, overexpression of HSPs in cancer cells aids their survival, thus contributing to disease progression.

Despite the established individual effectiveness of AR and HT and the known role of HSPs in cancer, the collective impact of these factors, particularly on human gastric adenocarcinoma (AGS) cells, is not well understood. Understanding the mechanisms underlying the potential synergistic effects of AR, HT, and HSPs could provide valuable insights into more effective cancer treatments. In this study, we explore the combined effects of AR and 43 °C HT on AGS cancer cell proliferation, apoptosis, and the role of HSPs and ROS in these processes. Based on the bioactive properties of costunolide and dehydrocostus lactone and their roles in ROS generation, we hypothesized that these compounds could significantly contribute to the observed therapeutic effects of the combined treatment. We also investigated the role of HSPs in this context, particularly in response to co-treatment with AR and HT. These findings could help pave the way for future research on the potential benefits of combined herbal and HT therapies in cancer treatment, considering the role of HSPs and ROS.

## 2. Materials and Methods

### 2.1. Preparation of AR Extract

AR (Kwangmyeongdang Medicinal Herbs Co. Ltd., Ulsan, Republic of Korea) was ground, extracted for 24 h at room temperature with 1 L of 70% EtOH, and filtered (pore size: 5 μm) using vacuum concentration and lyophilization. Prior to use, the drugs were kept at 4 °C, and dimethyl sulfoxide (DMSO) (Samcheon Chemical, Seoul, Republic of Korea) was used to produce concentrations of 10, 15, and 20 μg/mL.

### 2.2. Liquid Chromatography (LC)–Mass Spectrometry (MS) Analysis

Chemical components of AR were analyzed using ultra-performance liquid chromatography–electrospray ionization/quadrupole time-of-flight high-definition mass spectrometry (UPLC-ESI-QTOF-MS/MS). To prepare the sample, the extract was agitated for 30 s in 50% methanol using a vortex mixer, followed by 10 min of sonication. Subsequently, the supernatants were filtered through a 0.2 μm hydrophilic polytetrafluoroethylene syringe filter (Thermo Fisher Scientific, Sunnyvale, CA, USA). The filtered extract was then diluted to 100 mg/mL and transferred to an LC vial for analysis. The analysis was conducted using a Thermo Scientific Vanquish UHPLC system coupled with a Poreshell EC-C18 column (2.1 mm × 100 mm, 2.7 μm; Agilent, Santa Clara, CA, USA) and a Triple TOF5600+ mass spectrometer system (QTOF MS/MS, SCIEX, Foster City, CA, USA).

The QTOF MS system featured an electrospray ionization (ESI) source operable in both positive and negative ion modes for high-resolution experiments. The elution program for UHPLC involved 0.1% formic acid in water as eluent A and methanol as eluent B, progressing as follows: 0–10 min at 5% B; 10–30 min ramping from 5% to 80% B; 30–31 min from 80% to 100% B; 31–35 min at 100% B; and a 4 min equilibration at 5% B, with a flow rate of 0.3 mL/min. The column temperature was set at 25 °C and the autosampler temperature at 4 °C. Each sample was injected in a volume of 5 μL. Data for qualitative analysis were acquired and processed using Analyst TF 1.7, PeakView 2.2, and MasterView software 4.5 (SCIEX, Foster City, CA, USA), with MS/MS data further analyzed in PeakView and MasterView to identify probable metabolites based on accurate mass and isotopic distributions.

### 2.3. Cell Culture

AGS stomach cancer cells were obtained from the Korean Cell Line Bank (Seoul, Republic of Korea). The cells were maintained in an incubator at 37 °C with humidified air containing 5% CO_2_, in Dulbecco’s modified eagle medium (DMEM) supplemented with 10% fetal bovine serum (FBS; heat-inactivated) (Gibco, Grand Island, NY, USA) and 1% of Pen-Strep (10,000 U/mL) (Gibco, Grand Island, NY, USA).

### 2.4. HT Treatment

AR was applied to the samples at the designated concentrations, and after an hour, AGS cells (0.3 × 10⁶ cells/well) were seeded and suspended in 3 mL of media before being incubated in a water bath at 37 or 43 °C for 30 min.

### 2.5. MTT Assay

The 3-(4,5-dimethylthiazol-2-yl)-2,5-diphenyltetrazolium bromide (MTT) assay was used to confirm the synergistic effect of concurrent drug and heat treatment. AGS cells (1 × 10⁴ cells/mL) were plated on a 6-well plate, treated with 0, 10, 15, and 20 μg/mL of AR for an hour, and then exposed to 37 or 43 °C for 30 min. Twenty microliters per well of MTT reagent (2 mg/mL in phosphate-buffered saline; PBS) (AMRESCO, Solon, OH, USA) were added after 48 h of incubation. The wells were then incubated at 37 °C for 2 h in the dark. Following suction, 100 μL of DMSO was added to each well, the mixture was agitated, and the absorbance at 570 nm was determined using a spectrophotometric plate reader. Relative cell viability was standardized against untreated controls.

### 2.6. Trypan Blue Assay

Cell viability was determined using a trypan blue (Sigma-Aldrich, St. Louis, MO, USA) assay. After seeding, AGS cells (0.3 × 10⁶ cells/mL) were treated with AR at 20 μg/mL (control: 0 μg/mL AR) and allowed to react for 1 h, after which they were exposed to 37 or 43 °C for 30 min. After 24 h, the cells were collected, stained with a 1:4 diluted solution of 1 × PBS and trypan blue reagent, and counted using a hemocytometer. Cell survival was determined by

$$cell\;survival\;(\%)=\frac{viable\;cell\;count\;}{total\;cell\;count}\times 100\;$$

### 2.7. Morphology Assay

A morphology assay was performed to assess cell proliferation. AGS cells were seeded in a 6-well plate (0.3 × 10⁶ cells per well). After adherence, the cells were treated with 20 μg/mL AR for 1 h, and then incubated at 37 or 43 °C for an additional 30 min. Twenty-four hours later, cells were analyzed and imaged using an Olympus CX-40 microscope (Olympus, Tokyo, Japan).

### 2.8. Wound Healing Assay

AGS cells were seeded in a 6-well plate (0.3 × 10⁶ cells/well) and then cultured at 37 °C in an incubator. A thin scratch was made in each well with a micropipette tip, and the wells were treated with AR (20 μg/mL) and HT. Images were obtained using an Olympus CX-40 microscope (Olympus, Tokyo, Japan) at 0 and 24 h

### 2.9. Colony Formation Assay

Each well of a 6-well plate was filled with 400 cells, and the plates were incubated overnight. The cells were treated with 20 μg/mL AR for an hour after a 30 min incubation period at 37 or 43 °C. After two weeks, the cells were washed with 1 × PBS and stained with a crystal violet solution (Sigma-Aldrich, St. Louis, MO, USA) for 10 min at room temperature. An Olympus CX-40 microscope (Olympus, Tokyo, Japan) was used to examine the colonies.

### 2.10. Western Blot Analysis

Western blot analysis was performed as previously described [9]. Primary antibodies of anti-caspase-3, anti-caspase-8, anti-caspase-9, anti-p-ERK (Thr202/Tyr204), anti-ERK, anti-p-JNK (Thr183/Tyr185), anti-JNK, anti-p-p38 (Thr180/Tyr182), anti-p38, anti-HSP27, anti-HSP70, anti-HSP90 (Cell Signaling Technology, Danvers, MA, USA), anti-β-actin, anti-Bcl-2, anti- Bcl-xL, anti-Cyclin B1, anti-Cyclin D1, anti-MMP2, anti-MMP9, anti-VEGF (Santa Cruz Biotechnology, Inc., Dallas, TX, USA), and anti-cleaved caspase-3 (GeneTex) were used. Membranes were visualized using enhanced chemiluminescence (ECL) (Millipore, Billerica, MA, USA).

### 2.11. Apoptosis Assay

The apoptosis assay was performed using an Annexin V-fluorescein isothiocyanate (FITC) Detection Kit (ApoScan kit, Cat. No.: LS-02-100). AGS cells (0.3 × 10⁶ cells/well in a 6-well plate) were treated with 0 and 20 μg/mL concentrations of AR for 1 h, followed by exposure to temperatures of 37 °C or 43 °C for 30 min. After 24 h, the cells were harvested and stained with Annexin V-FITC for 15 min at room temperature in the dark, as per the manufacturer’s instructions. Subsequent to staining, the cells underwent centrifugation to discard the supernatant, and propidium iodide (PI) staining was applied using 1× cold binding buffer. Apoptosis levels were quantified via flow cytometry.

### 2.12. Cell Cycle Analysis

AGS cells (0.3 × 10⁶ cells/well in 6-well plates) treated simultaneously with AR and HT for 24 h for cell cycle analysis. Then, the cells were collected, fixed in 70% ethanol for 24 h, and subsequently washed with 1× cold PBS. The cells were then resuspended in PBS containing 1 mg/mL PI and 10 mg/mL RNase A, and incubated in the dark for 10 min. Cell cycles were assessed using a flow cytometer.

### 2.13. Analysis of ROS

H2DCFDA (2′,7′-dichlorofluorescin diacetate or InvitrogenTM D399; Thermo Fisher Scientific) reagent was used to measure cellular ROS. After seeding the AGS cells (0.3 × 10⁶ cells/well), they were co-treated and then incubated. After 4 h, the cells were harvested to obtain a pellet, and 10 μM H2DCFDA reagent was added, followed by a 40 min incubation at 37 °C in the dark. Flow cytometry was used to calculate ROS levels.

### 2.14. Statistical Analysis

The mean ± SD is used to represent all numerical values. Statistical significance was determined using Student’s unpaired *t*-tests. * *p* < 0.05, ** *p* < 0.01 and *** *p* < 0.001.

## 3. Results

### 3.1. Chemical Components of AR Identified by UPLC-ESI-QTOF-MS/MS

**Figure 1:**
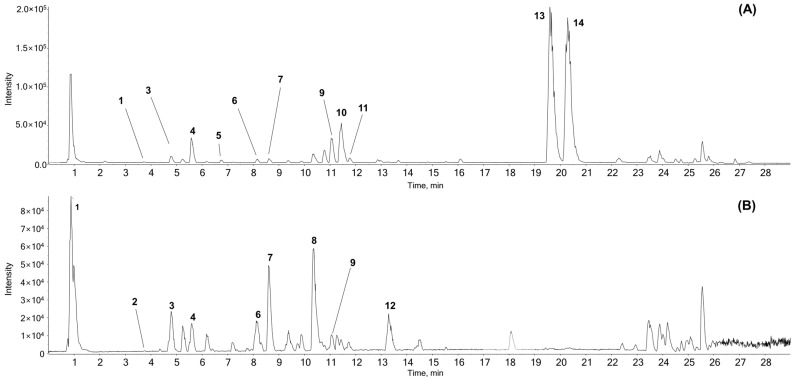
Representative base peak chromatogram of AR using UPLC-ESI-QTOF MS/MS analysis in (A) positive and (B) negative ion modes. AR, Aucklandiae Radix; UPLC-ESI-QTOF MS/MS, ultra-performance liquid chromatography-electrospray ionization/quadrupole time-of-flight mass spectrometry/mass spectrometry.

The ethanolic extract was analyzed by UPLC-ESI-QTOF-MS/MS analysis to determine the chemical profile of its constituents. The extracted ion chromatogram identified 14 components including costunolide (**13**) and dehydrocostus lactone (**14**), which are the two main compounds in AR (Figure 1). Area under curve shows that costunolide (**13**) accounts for 9.74% and dehydrocostus lactone (**14**) accounts for 10.02%. Of note, 3,4-dicaffeoylquinic acid (**7**) accounted for 0.40%, parthenolide (**11**) for 0.36%, tryptophan (**2**) for 0.07%, syringaldehyde (**5**) for 0.03%. Detailed information of the detected peaks is listed in Table 1.

### 3.2. Co-Treatment with AR and 43 °C HT Synergistically Inhibited AGS Cell Proliferation

**Figure 2:**
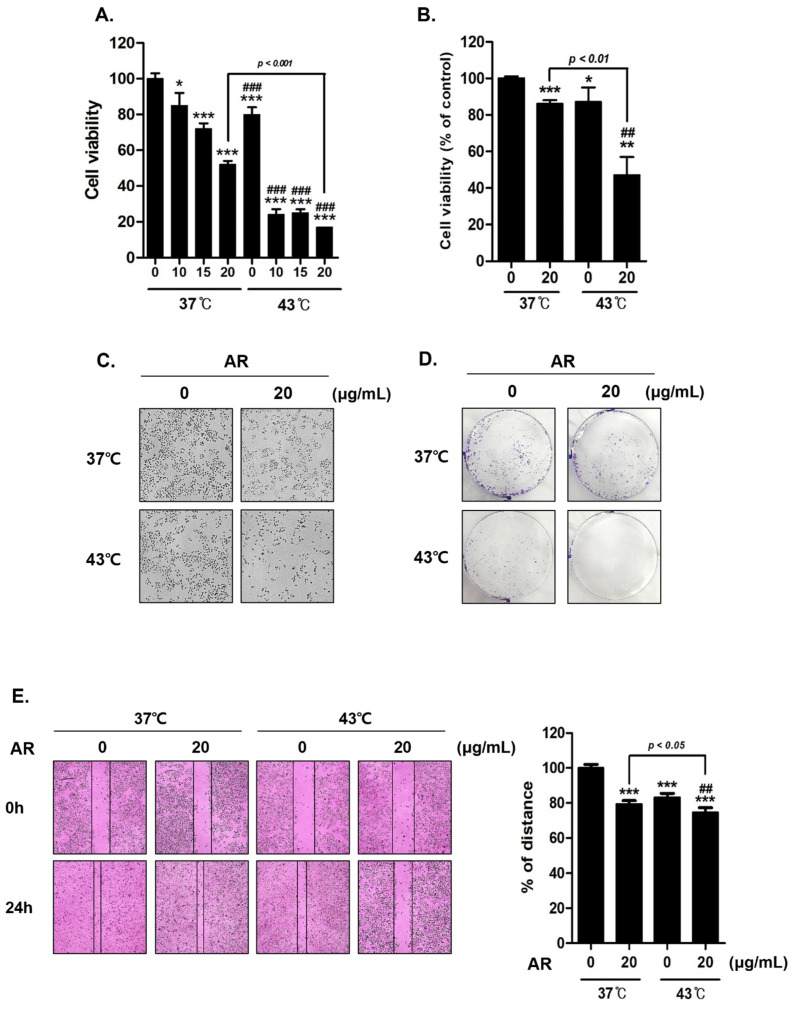
Effect of AR and HT on AGS cell viability. AGS cells were treated with various concentrations of AR (0, 10, 15, and 20 μg/mL) for 24 h with (at 43 °C) or without (at 37 °C) HT. (**A**) Percentage cell viability was determined using the MTT assay. (**B**) The trypan blue staining was performed to determine live cell percentage under a regular light microscope. (**C**) Apoptosis-related morphological changes as seen at 100× magnification. (**D**) For the clonogenic experiment, crystal violet staining was utilized. (**E**) Wound healing assays were performed to determine changes in cell migration of control and treated cells at 0 and 24 h. Bar graphs indicate the gap distance percentage at hour 24 compared to hour 0. * *p* < 0.05, ** *p* < 0.01, *** *p* < 0.001 vs. control group; ## *p* < 0.01, ### *p* < 0.001 vs. 43 °C + 0 μg/mL group.

The combination of AR and 43 °C exhibited a synergistic effect in hampering the proliferation of AGS cells. To test the efficacy of the treatments, we conducted MTT assays. When subjected to an identical dose of AR (20 µg/mL), we observed that AGS cell viability was reduced more notably by the combined treatment with 43 °C HT than the one with 37 °C (Figure 2A). This effect of the combination of AR and HT was further substantiated by trypan blue staining of surviving cells (Figure 2B) and an apparent alteration in cell morphology (Figure 2C). Upon evaluation of crystal violet-stained AGS cells, the combination treatment with AR and 43 °C resulted in a significant drop in colony formation relative to the treatment involving AR and 37 °C (Figure 2D). The subsequent cell migration analysis revealed that the combination of AR and HT inhibited cell migration (Figure 2E).

### 3.3. The Combined Application of AR and 43 °C HT Enhanced the Manifestation of Factors Associated with Apoptosis and Suppressed Factors Related to Protection and Proliferation in AGS Cells

**Figure 3:**
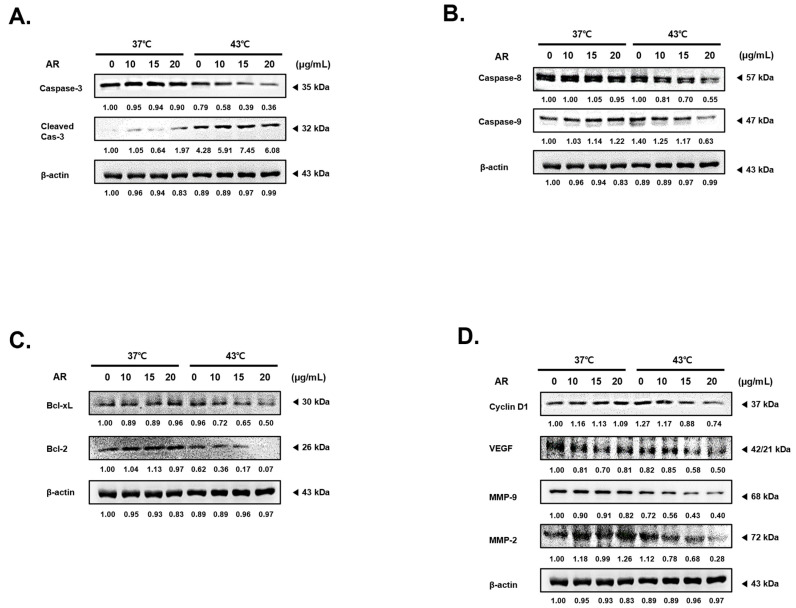
Effect of AR and HT on survival, proliferation, and angiogenesis expression levels in the treatment and control groups. The protein expressions of (**A**) caspase-3, (**B**) caspase-8, caspase-9, (**C**) Bcl-xL, Bcl-2, and (**D**) Cyclin D1, VEGF, MMP-9, and MMP-2 were determined by Western blot. β-actin was used as a loading control. Bcl, B-cell lymphoma; xL, extra large; VEGF, vascular endothelial growth factor; MMP, matrix metallopeptidase.

In the next phase of our research, we validated the expression levels of factors associated with processes such as apoptosis, cell proliferation, metastasis/angiogenesis to elucidate the synergistic mechanism triggered by concurrent treatment with AR and HT. As depicted in Figure 3A, a combined treatment with AR and HT at 43 °C significantly promoted the cleavage of caspase-3, which signifies the culmination of programmed apoptosis [37]. This phenomenon was not evident when AR was paired with an HT of 37 °C. We assessed caspase-8 and caspase-9, which are the primary caspases associated with extrinsic and intrinsic apoptosis, respectively (Figure 3B) [38]. In accordance with the findings related to cleaved caspase-3, caspase-8 and -9 expression decreased in a dose-dependent manner, but only when subjected to a combined treatment with AR and 43 °C HT. Furthermore, concurrently treating with AR and 43 °C HT led to a dose-dependent decrease in the expression levels of anti-apoptotic constituents of the B-cell lymphoma (Bcl)-2 family, which included Bcl-2 and Bcl-xL (Bcl-extra large) (Figure 3C) [39]. Our study also examined Cyclin D1 [40], a modulator of cell adhesion and migration linked to cancer cell invasion and metastasis; vascular endothelial growth factor (VEGF) [41], a central molecule in angiogenesis; and matrix metallopeptidase (MMP)-9, an instrumental member of the MMP family implicated in tumor metastasis [42]. Our data unequivocally demonstrated that these metastatic factors were curtailed by the combination of AR and 43 °C HT (Figure 3D).

### 3.4. The Combined Application of AR and 43 °C HT Induced Apoptosis and Cell Cycle Arrest in AGS Cells

**Figure 4:**
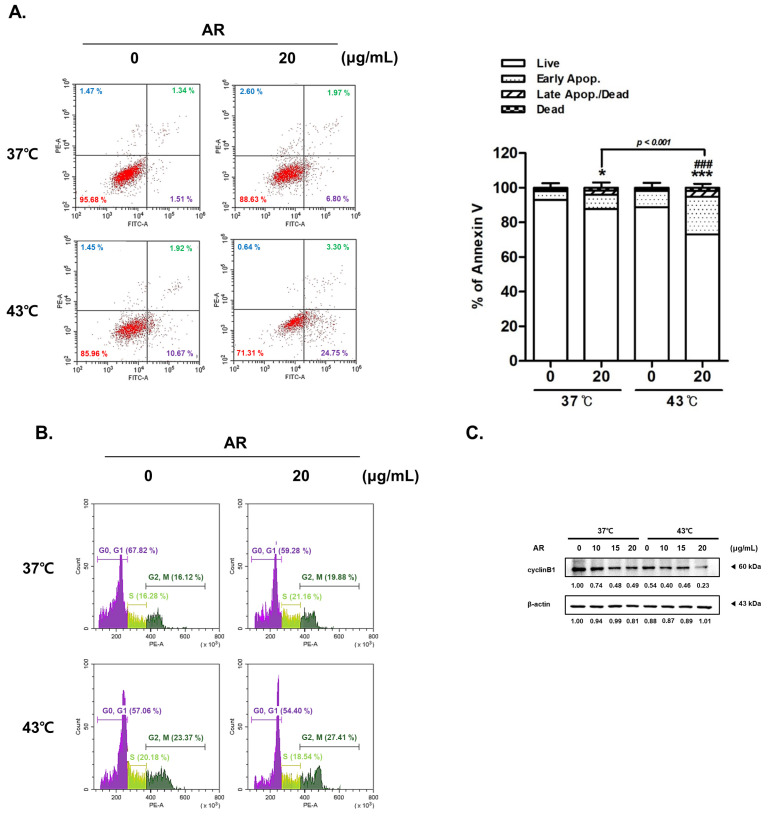
Effect of AR and HT on the cell cycle and apoptosis in AGS cells. AR (0 or 20 μg/mL) was applied to AGS cells (0.3 × 10⁶ cells) with or without HT. Flow cytometry was used to determine apoptosis with Annexin V and PI staining. (**A**) Apoptosis profile and (**B**) cell cycle profile. (**C**) A Western blot assay was used to gauge Cyclin B1 expression. A loading control was performed with β-actin. Apop., apoptosis. * *p* < 0.05, *** *p* < 0.001 vs. control group; ### *p* < 0.001 vs. 43 °C + 0 μg/mL group.

As demonstrated in Figure 4A, the combined treatment with AR and 43 °C HT increased apoptosis linked to Annexin V in AGS cells, and notably more than the 43 °C HT on its own or AR paired with normothermia. The cooperative influence of AR and HT was dose-dependent; the proportion of apoptotic cells increased from 2.85% (0 µg/mL AR, 37 °C) through 8.77% (20 µg/mL AR, 37 °C) and 12.59% (0 µg/mL AR, 43 °C) to 28.05% (20 µg/mL AR, 43 °C).

Cell cycle arrest is closely associated with the induction of apoptosis [43]. This process is a common therapeutic target for cancer treatment [44]. Flow cytometry was performed to establish whether the combined treatment with AR and HT had any effect on cell cycle arrest. We found that the combined treatment with AR and 43 °C HT halted the cell cycle in the gap 2/mitosis (G2/M) phase (Figure 4B) [45]. The treatment with AR in conjunction with 43 °C HT also significantly diminished cyclin B1 expression, thereby supporting the hypothesis that the co-treatment of AR and HT induces cell cycle arrest in AGS cells during the G2/M phase (Figure 4C). Taken together, these findings provide compelling evidence of the potential benefits of combining AR with 43 °C HT in inducing apoptosis and arresting the cell cycle in AGS cells, thereby providing novel insights into promising therapeutic approaches for cancer treatment.

### 3.5. The Combined Application of AR and 43 °C HT Synergistically Inhibited HSP Expression, Leading to ROS-Dependent Apoptosis

**Figure 5:**
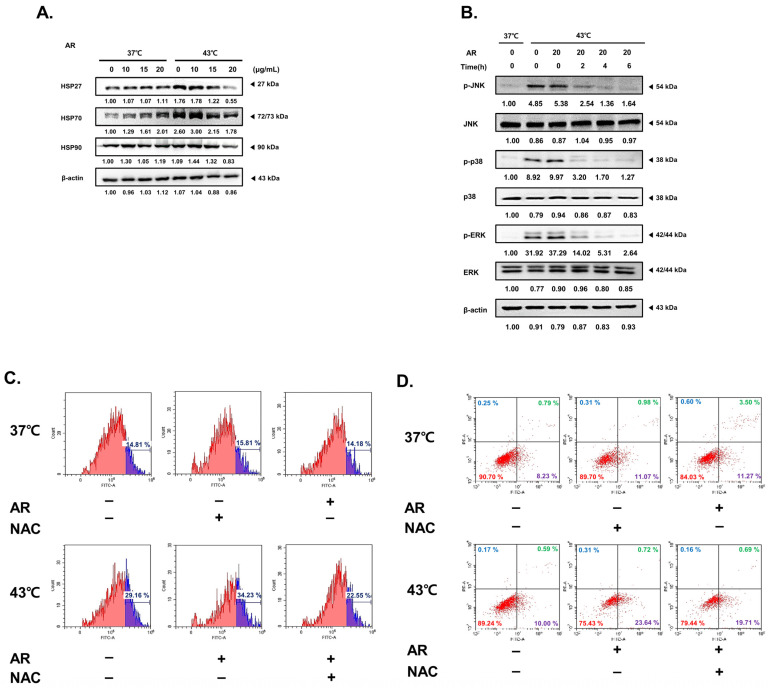
Effect of co-treatment with AR and HT on the mitogen-activated protein kinase (MAPK) pathway, apoptotic markers, and ROS production in ROS-inhibited AGS cells. AGS cells were pre-treated with N-acetylcysteine (NAC) (5 mM) for 1 h before being exposed to AR (0 or 20 μg/mL) with (at 43 °C) or without (at 37 °C) HT. (**A**) HSP27, HSP70, and HSP90 expression. (**B**) Western blot assays were used to measure the concentrations of phosphor (p)-Jun N-terminal kinase (JNK), JNK, p-p38, p38, p-extracellular signal-regulated kinase (ERK), and ERK. (**C**) Flow cytometry was used to investigate ROS generation. (**D**) Apoptosis profiling was carried out in the presence of NAC or AR (+) and absence of NAC or AR (−).

HSPs are known for their role in eliminating appropriate amounts of ROS from cancer cells, thereby facilitating their growth [46]. Therefore, we investigated how the combined application of AR and 43 °C HT could influence HSP expression and ROS levels. As in Figure 5A, HSPs 27, 70, and 90 were increased in AGS cells upon incuabtion at 43 °C HT, as expected. Treatment with AR markedly decreased HSP expression under HT. Considering the decreased expression of HSPs, co-treatment can augment ROS levels, which can activate mitogen-activated protein kinases (MAPKs), leading to the consequent induction of apoptosis [47]. As shown in Figure 5B, co-treatment boosted the phosphorylation of MAPKs, including Jun N-terminal kinase (JNK), p38, and extracellular signal-regulated kinase (ERK), at 43 °C HT. We also observed that co-treatment increased ROS production and decreased ROS levels following N-acetyl-L-cystein (NAC) pretreatment. Subsequently, we conducted an experiment to establish the correlation between ROS production (Figure 5C) and the effectiveness of co-treatment. As shown in Figure 5D, the co-treatment demonstrated a synergistic effect, inducing apoptosis in cancer cells. However, NAC pretreatment mitigated this effect. Without the ROS signaling, combination treatment with AR and HT fail to interrupt the self-protection system induced by heat shock.

### 3.6. ROS Scavenging Inhibited the Apoptotic Effect of the Combined AR and HT Treatment and Recovered HSP Expression

**Figure 6:**
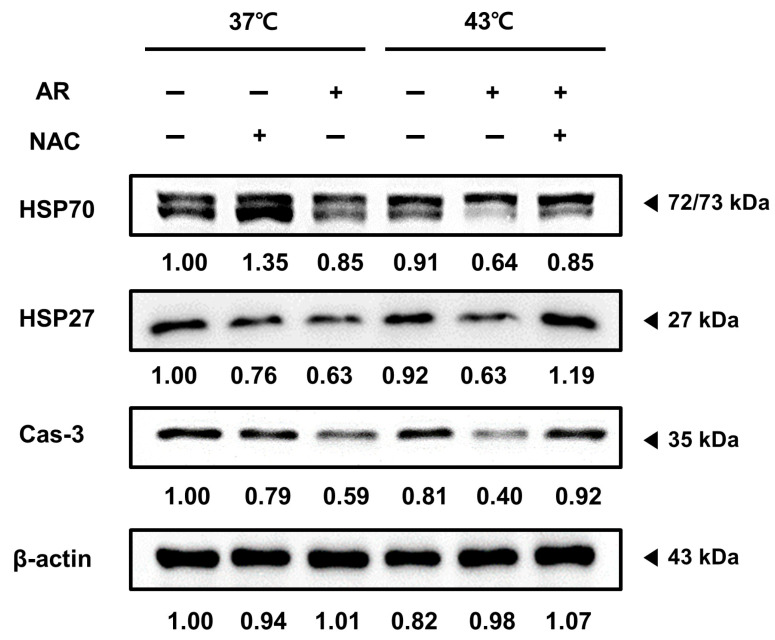
Effect of co-treatment in ROS-inhibited AGS cells reduced apoptosis and increased HSP expression. Expressions of the proteins HSP70, HSP27, and caspase-3 in the presence of NAC or AR (+) and absence of NAC or AR (−). AGS cells (0.3 × 10⁶) were pre-treated with NAC (5 mM) for 1 h before being exposed to AR with (at 43 °C) or without (at 37 °C) HT.

Next, we investigated whether NAC pretreatment could reverse the suppressed expression of HSP70, HSP27, and caspase-3 induced by the AR and HT co-treatment, suggesting that the co-treatment effects depended on ROS generation. Figure 6 shows that NAC pretreatment reversed the changes in expression of HSP70, HSP27, and caspase-3 induced by the co-treatment, indicating that the co-treatment effects were dependent, at least partly, on ROS and HSP.

## 4. Discussion

Our study sought to investigate the synergistic effects of AR and HT at 43 °C on inhibiting AGS cancer cell proliferation and promoting apoptosis. The herbal extract AR, which is known for its rich costunolide and dehydrocostus lactone content, has been associated with significant anti-cancer activities [48,49,50]. To our knowledge, this is the first research that explores the synergistic effects of AR and 43 °C HT on cancer cells.

In our study, costunolide and dehydrocostus lactone, the two primary components of AR, drew significant attention. Both compounds are known for their broad-spectrum biological activities such as anti-inflammatory, anti-microbial, anti-ulcer, and anti-cancer features [51,52,53,54]. Notably, the mechanisms underlying their anti-cancer effects include inducing ROS and promoting apoptosis, which reflects our current findings [55,56,57]. Therefore, these two compounds may be the critical drivers of the synergistic effects observed in our study.

Our results demonstrated that the combination of AR and 43 °C HT led to a significant decrease in the viability and proliferation of AGS cells, pointing to the effectiveness of the treatment against cancer. These findings were further substantiated by the observable alterations in cell morphology, reduced cell migration, and diminished colony formation. We observed an enhanced induction of apoptosis under the combined treatment of AR and 43 °C HT, as evidenced by the increased cleavage of caspase-3, a key factor in programmed cell death [58]. This trend was mirrored in the levels of caspase-8 and caspase-9, which decreased under the combined treatment. Interestingly, this dose-dependent response was unique to the combined treatment, highlighting the distinctive potency of this approach.

Furthermore, this co-treatment induced apoptosis and suppressed anti-apoptotic factors Bcl-2 and Bcl-xL [59]. This dual action likely intensifies the apoptotic effects in AGS cells. This study also sheds light on the downregulation of factors related to metastasis and angiogenesis, such as Cyclin D1, VEGF, and MMP-9, under combined treatment, emphasizing the potential therapeutic promise of this strategy for controlling cancer cell proliferation and metastasis [60,61,62,63]. Importantly, the combined application of AR and 43 °C HT was found to induce cell cycle arrest at the G2/M phase, a process closely associated with the provocation of apoptosis [64,65]. Our findings suggest that the occurrence of apoptosis was dose-dependent under the combined treatment, suggesting the potential for a precise therapeutic approach.

Our study also revealed the critical role of ROS in the apoptotic effects of the combined treatment. HSPs, which are often linked to cell survival under stress, were inhibited by the combined treatment, resulting in increased ROS levels, which subsequently induced apoptosis. Notably, costunolide and dehydrocostus lactone have been reported to promote the generation of ROS, thereby enhancing oxidative stress and driving cancer cells toward apoptosis [66,67]. Thus, our findings are consistent with these previous reports, indicating that the ROS-mediated mechanism could be one of the key pathways involved in the synergistic anti-cancer effects of the combined treatment. However, when ROS were scavenged using NAC, a reversal of the apoptotic effect and recovery of HSP expression were observed, emphasizing the essential role of ROS in the desired therapeutic effects and indicating the potential limitations of this therapy in cancer cells with strong ROS-scavenging abilities.

**Figure 7:**
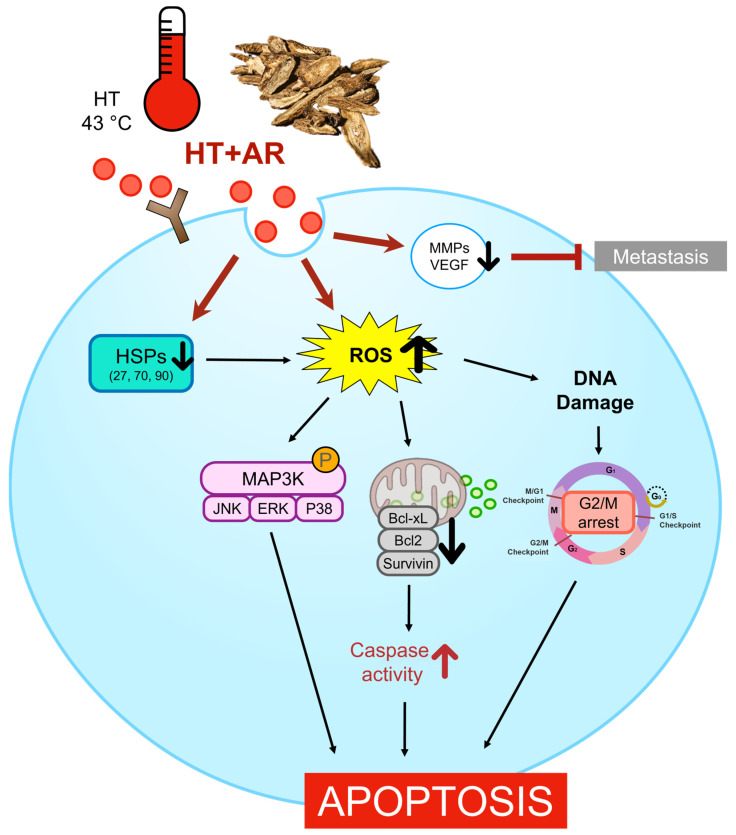
Schematic of the present study. Combination treatment with AR and 43 °C HT induces ROS-dependent intrinsic apoptosis in AGS cells. While HSF and HSP expressions are increased by HT, AR co-treatment suppresses the protective program in response to heat. AR and HT combination also induces cell cycle arrest, decreases expressions of MMPs and VEGF, and suppresses MAPK activation.

In conclusion, our study offers compelling evidence to support the synergistic anti-cancer potential of AR and HT at 43 °C on AGS cells, mainly through promoting intrinsic apoptosis, inhibiting cell proliferation, and inducing cell cycle arrest (Figure 7). In particular, the presence of compounds such as costunolide and dehydrocostus lactone, which are known for their anti-cancer activities and roles in promoting ROS, likely enhances the effectiveness of combined AR and HT treatment [68,69,70]. These findings suggest that with the beneficial effects of costunolide and dehydrocostus lactone, the combination of AR and HT could emerge as a promising therapeutic approach for cancer treatment. However, future research should aim to prove this potential anti-cancer effects of AR and HT co-treatment in clinical settings.

## Figures and Tables

**Table 1:** Detected peak list from the UPLC-ESI-QTOF-MS/MS analysis of AR.

No.	Name	Formula	Mass (Da)	Expected	Adduct	Found at	Error	MS/MS Product ions	Identified with
				RT (min)		Mass (Da)	(ppm)		
1	Citric acid	C_6_H_8_O_7_	192.027	1.05	[M − H]^−^	191.0199	0.9	111.0101, 87.01303, 85.0309	^#^
2	Tryptophan	C_11_H_12_N_2_O_2_	204.08988	3.73	[M + H]^+^	205.09696	−1	146.0593, 118.0656, 188.0686, 143.0728, 144.0801	^#^
					[M − H]^−^	203.08269	0.5	116.0497, 142.0670,74.0270	
3	Chlorogenic acid	C_16_H_18_O_9_	354.09508	4.72	[M + H]^+^	355.1026	0.7	163.0384, 145.0276, 135.0435, 117.0332	^#^
					[M − H]^−^	353.08777	−0.1	191.0557, 161.0237, 173.0439	
4	Unknown	C_26_H_39_NO_10_	525.2574	5.6	[M + H]^+^	526.26426	−0.8	364.2122, 128.0708, 346.2013	^*^
					[M − H]^−^	524.24949	−1.2	114.0566, 209.1889, 247.1327	
5	Syringaldehyde	C_9_H_10_O_4_	182.05791	6.83	[M + H]^+^	183.0651	−0.6	77.0402, 95.0497, 140.0465, 123.0442	^#^
6	1,5-Dicaffeoylquinic acid	C_25_H_24_O_12_	516.12678	8.15	[M + H]^+^	517.13348	−1.1	163.0386, 145.0280, 135.0445, 319.0813	^#^
					[M − H]^−^	515.11915	−0.7	191.0558, 353.0873, 179.0341, 161.0245	
7	3,4-Dicaffeoylquinic acid	C_25_H_24_O_12_	516.12678	8.63	[M + H]^+^	517.13357	−0.9	163.0384, 145.0289, 135.0445, 319.0809	^#^
					[M − H]^−^	515.11901	−0.9	353.0876, 173.0451, 179.0346, 191.0551	
8	Unknown	C_22_H_32_O_10_	456.19955	10.35	[M − H]^−^	455.1917	−1.2	247.1333, 409.1877, 203.1431, 135.0809	*
9	Unknown	C_20_H_27_NO_4_	345.19401	11.07	[M + H]^+^	346.20148	0.5	300.1963, 128.0708, 100.0766, 88.0668, 70.0671	^*^
					[M − H]^−^	344.18673	0	114.0572, 113.0690	
10	Unknown	C_21_H_28_N_2_O	324.22016	11.43	[M + H]^+^	325.22767	0.7	91.0560, 86.0983, 233.1653, 84.0825	*
11	Parthenolide	C_15_H_20_O_3_	248.14124	11.77	[M + H]^+^	249.14857	0.6	185.1325, 143.0856, 128.0617, 129.0696	^#^
12	Unknown	C_18_H_34_O_5_	330.24062	13.33	[M − H]^−^	329.23305	−0.9	211.1342, 229.1444, 171.1028, 139.1135	*
13	Costunolide^†^	C_15_H_20_O_2_	232.14633	19.63	[M + H]^+^	233.15364	0.2	187.1482, 145.1017, 131.0854, 105.0708, 91.0554	*
14	Dehydrocostuslactone^†^	C_15_H_18_O_2_	230.13068	20.30	[M + H]^+^	231.13814	0.8	143.0861, 128.0624, 129.0705, 185.1332	^#^

# In-house ms/ms library and online database; such as GNPS, MASS bank or Metlin. † Reference standard, * Extract MS with isotope mass.

## Data Availability

The data presented in this study are available on request from the corresponding authors.
